# TGF-β3 modulates the inflammatory environment and reduces scar formation following vocal fold mucosal injury in rats

**DOI:** 10.1242/dmm.013326

**Published:** 2013-10-02

**Authors:** Zhen Chang, Yo Kishimoto, Ayesha Hasan, Nathan V. Welham

**Affiliations:** Department of Surgery, Division of Otolaryngology, University of Wisconsin School of Medicine and Public Health, Madison, WI, USA.

**Keywords:** Fibrosis, Larynx, Tissue repair, Wound healing

## Abstract

Transforming growth factor (TGF)-β1 and TGF-β3 have been reported to exert differential effects on wound healing, and possibly even account for tissue-specific differences in scar formation. Scarring is particularly detrimental in the vocal fold mucosa (VFM), where destruction of the native extracellular matrix causes irreparable biomechanical changes and voice impairment. Here, in a series of *in vitro* and *in vivo* experiments, we identified differences in TGF-β1 and TGF-β3 transcription and immunolocalization to various cell subpopulations in naïve and injured rat VFM, compared with oral mucosa (which undergoes rapid healing with minimal scar) and skin (which typically heals with scar). Treatment of cultured human vocal fold fibroblasts with TGF-β3 resulted in less potent induction of profibrotic gene transcription, extracellular matrix synthesis and fibroblast-myofibroblast differentiation, compared with treatment with TGF-β1 and TGF-β2. Finally, delivery of exogenous TGF-β3 to rat VFM during the acute injury phase modulated the early inflammatory environment and reduced eventual scar formation. These experiments show that the TGF-β isoforms have distinct roles in VFM maintenance and repair, and that TGF-β3 redirects wound healing to improve VFM scar outcomes *in vivo*.

## INTRODUCTION

Vocal fold mucosal (VFM) injury, scar formation and its resultant dysphonia represent a significant clinical problem with no robust therapeutic options (Welham et al., 2011). Similar to other tissues, acute VFM injury initiates a tissue repair process consisting of overlapping cellular events including inflammation, proliferation and reepithelialization, contraction, extracellular matrix (ECM) synthesis and remodeling (Branski et al., 2006; Gurtner et al., 2008; Ling et al., 2010). The manner in which these events initiate, proceed and terminate has a direct effect on eventual wound healing outcome. For example, although platelet, neutrophil and macrophage infiltration is necessary to prevent blood loss and infection, remove cell debris and deliver key signaling molecules, excessive and/or prolonged inflammation can lead to fibrosis (Martin and Leibovich, 2005; Stramer et al., 2007). Similarly, fibroblast activation and differentiation to a contractile myofibroblast phenotype is important for wound closure and ECM deposition; however, if this tissue repair phase does not properly terminate, persistent myofibroblast activation can lead to excessive wound contraction and ECM deposition, again culminating in a fibrotic outcome (Gurtner et al., 2008; Serini and Gabbiana, 1996).

The transforming growth factor-β (TGF-β) superfamily plays a key regulatory role in these tissue repair processes (O’Kane and Ferguson, 1997) and evidence suggests that individual TGF-β isoforms differentially affect wound healing outcomes. Specifically, TGF-β1 and TGF-β2 are associated with fibrotic healing in adult wounds, whereas TGF-β3 is associated with regenerative healing in fetal wounds (Ferguson and O’Kane, 2004; Occleston et al., 2011; Shah et al., 1995; Whitby and Ferguson, 1991). Delivery of recombinant or therapeutically mutated TGF-β3 at the time of injury has been shown to reduce scar formation in skin (Shah et al., 1995; Waddington et al., 2010), lip (Hosokawa et al., 2003), oral mucosa (OM) (Ohno et al., 2011) and VFM (Ohno et al., 2012) in preclinical models, and recombinant TGF-β3 has demonstrated safety and efficacy in phase I and II human clinical trials (Ferguson et al., 2009; McCollum et al., 2011; So et al., 2011). Despite these promising outcomes, the mechanism of TGF-β3 action following injury is unclear, particularly with respect to cross-tissue variation in scar formation. It has been proposed that TGF-β3 modulates multiple tissue repair events including the inflammatory response, cell migration, fibroblast-myofibroblast differentiation, and ECM synthesis and organization (Bandyopadhyay et al., 2006; Murata et al., 1997; Occleston et al., 2008b; Occleston et al., 2011; Serini and Gabbiana, 1996). To date, although the temporal regulation of TGF-β1 transcription following VFM injury has been reported (Lim et al., 2006; Ohno et al., 2009), there are no VFM-specific TGF-β3 mechanistic data available, and so it is unknown whether TGF-β3 is constitutively expressed by cells within the VFM, whether endogenous production occurs during normal tissue repair, how exogenous delivery modulates tissue repair outcome and how these biologic events compare to those in non-VF mucosae and skin.

In this study, we evaluated the transcription and immunolocalization of TGF-β1 and TGF-β3 in naïve and injured rat VFM, compared with OM (which undergoes rapid healing with minimal scar) and skin (which typically heals with scar) (Schrementi et al., 2008). We then used cell culture experiments to analyze the capacity of exogenous TGFs β1, β2 and β3 to regulate profibrotic gene transcription, ECM synthesis, and fibroblast-myofibroblast differentiation in human vocal fold fibroblasts (VFFs). Finally, we evaluated the therapeutic potential of TGF-β3 to modulate the early inflammatory environment and eventual scar outcome in an established rat VFM injury model.

## RESULTS

### Immunohistochemical localization of TGF-β1 and TGF-β3 in naïve rat VFM

**Fig. 1:**
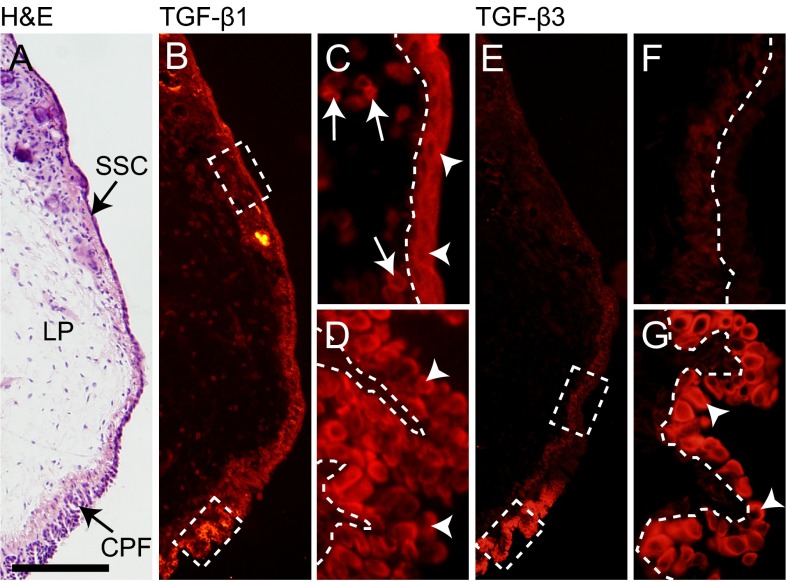
**Differential immunoactivity of TGF-β1 and TGF-β3 in naïve rat VFM.** (A) Representative H&E-stained coronal section illustrating the lamina propria (LP), stratified squamous cells (SSC) along the superior aspect of the epithelium, and ciliated pseudocolumnar cell formation (CPF) along the inferior aspect of the epithelium. (B) Representative image showing positive immunostaining for TGF-β1 (red). (C,D) Enlarged images of the regions indicated by dashed boxes in B. Arrows indicate TGF-β1^+^ LP cells. Arrowheads indicate TGF-β1^+^ epithelial cells in the SSC and CPF regions. (E) Representative image showing positive immunostaining for TGF-β3 (red). (F,G) Enlarged images of the regions indicated by dashed boxes in E. Arrowheads indicate TGF-β3^+^ epithelial cells in the CPF region. Each image is representative of three independent animals and >3 replicate sections per animal. Dashed lines in C,D,F,G indicate the boundary between the LP (left) and epithelium (right). Scale bar: 300 μm (A,B,E); 30 μm (C,D,F,G).

VFM is a layered structure comprised of lamina propria (LP) and epithelium, which in the inferior region transitions from stratified squamous cell (SSC) to ciliated pseudocolumnar cell formation (CPF) (Fig. 1A). Given the lack of data on TGF-β isoform expression and localization in VFM, we used isoform-specific antibodies (Flanders et al., 1989; Flanders et al., 1991) to characterize basal TGF-β1 and TGF-β3 expression by VFM cell subpopulations, prior to evaluating their role in the injury response. Immunohistochemical (IHC) staining revealed differential TGF-β1 and TGF-β3 expression by tissue region (Fig. 1B–G). Whereas TGF-β1 was expressed by LP cells [the majority of which are VFFs (Catten et al., 1998)] and epithelial cells in both SSC and CPF regions (Fig. 1B–D), TGF-β3 was predominantly expressed by the CPF population (Fig. 1E,G).

 

**TRANSLATIONAL IMPACT**

**Clinical issue**

Injury to the vocal fold mucosa (VFM) initiates a series of wound healing events that can lead to scar formation. Scarred VFM is characterized by disordered extracellular matrix and vibratory capacity, which can result in loss of voice (dysphonia). There are currently no effective therapies for scarred VFM. The transforming growth factor-β (TGF-β) cytokines are well-documented regulators of inflammation, tissue repair and fibrosis, and evidence suggests that differences in the relative abundance of isoforms TGF-β1 and TGF-β3 during the early post-injury phase might direct the wound environment on a pathway towards either fibrotic or regenerative repair. Increasing levels of TGF-β3 at the time of injury has been shown to attenuate scar formation in a number of model systems; however, the mechanism of action of TGF-β3 following tissue injury remains unclear, particularly in the VFM.

**Results**

Using cultured human vocal fold fibroblasts and a previously validated *in vivo* rat model, the authors show that TGF-β1 and TGF-β3 are differentially expressed and localized to various cell populations within naïve and injured VFM, and in VFM compared with oral mucosa and skin. TGF-β1 is expressed throughout the VFM following injury, whereas TGF-β3 is transiently expressed during reepithelialization. Compared with TGF-β1 and TGF-β2, TGF-β3 acts as a less potent inducer of profibrotic molecule expression and fibroblast-myofibroblast differentiation. Furthermore, delivery of exogenous TGF-β3 during the acute injury phase modulates early inflammatory events and improves VFM healing *in vivo*.

**Implications and future directions**

The authors confirm a relationship between TGF-β isoform expression pattern and the severity of scarring in different tissues, and also demonstrate the potential of TGF-β3 for reducing VFM scarring and its resultant dysphonia. These findings suggest that this biologic agent might one day be useful as an adjunct therapy delivered at the time of surgery, which is one of the major causes of VFM injury. Further studies are needed to better understand the sequential relationship between TGF-β3-mediated redirection of early inflammatory events, fibrotic outcome and functional tissue performance, in addition to determining optimal therapeutic dosing and *in vivo* safety.

### Differential response of TGF-β1 and TGF-β3 to injury in VFM, OM and skin

**Fig. 2:**
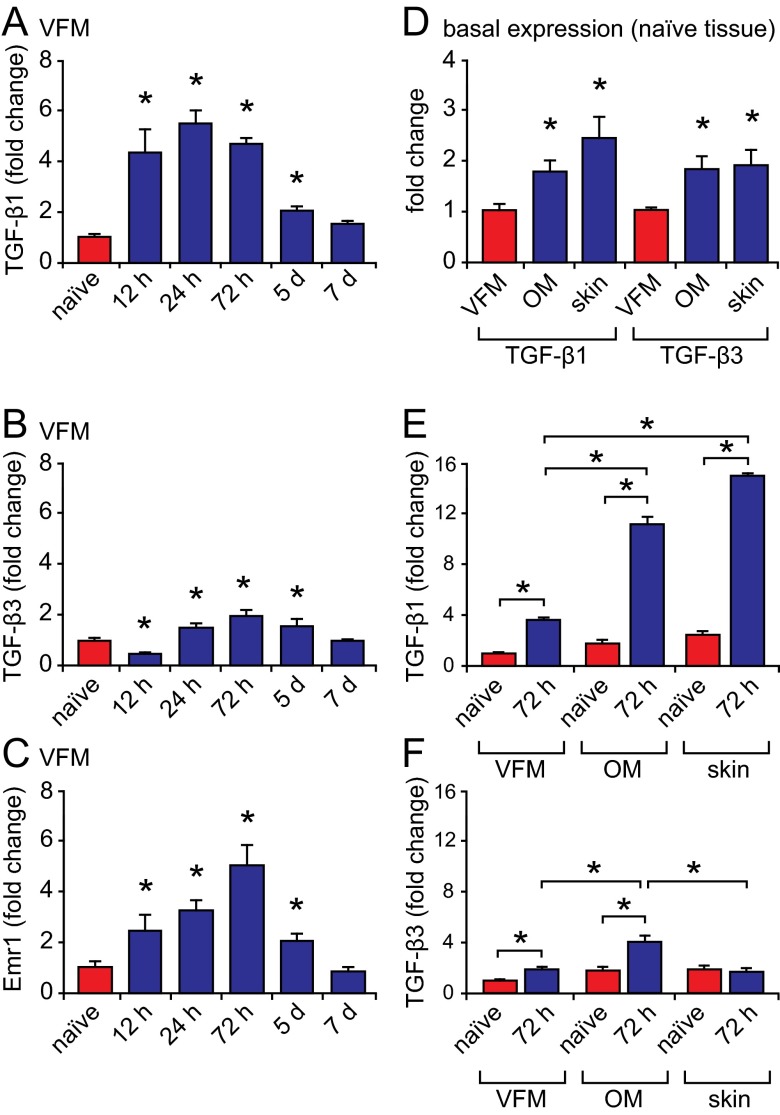
**TGF-β1 and TGF-β3 transcription in injured VFM, OM and skin.** (A-C) TGF-β1, TGF-β3 and macrophage-specific transcript Emr1 mRNA expression in VFM up to 7 days post-injury. (D) Comparison of basal TGF-β1 and TGF-β3 mRNA expression in naïve VFM, OM and skin. (E,F) Comparison of TGF-β1 and TGF-β3 mRNA expression in naïve and injured (72 hours post-injury) VFM, OM and skin. Results represent the means ± s.e.m. of three independent experiments with *n*=5 animals per time point, and are presented as fold change relative to naïve VFM. *P<0.01 versus naïve control within tissue type (A-C,E,F), versus naïve VFM (D) and across tissue types at 72 hours post-injury (E, F).

Having confirmed the presence of TGF-β1^+^ and TGF-β3^+^ cells in naïve VFM, we investigated the transcriptional profile of each isoform following acute injury, in VFM as well as OM and skin. We pursued these cross-tissue comparisons based on data indicating that injured OM heals with less TGF-β1 production and scar formation than injured skin (Schrementi et al., 2008). TGF-β1 transcription increased ~fourfold in VFM at 12 hours, peaked at 24 hours, and returned to naïve control levels by 7 days post-injury (*P*<0.01; Fig. 2A). Injury-induced TGF-β3 transcription was generally lower than that observed for TGF-β1: it decreased at 12 hours, increased to reach ~twofold peak expression at 72 hours, and returned to naïve control levels by 7 days post-injury (*P*<0.01; Fig. 2B). Parallel evaluation of the macrophage-specific (and F4/80 homolog) transcript EGF-like module-containing mucin-like hormone receptor-like 1 (Emr1) (Hamann, 2004; McKnight et al., 1996) revealed a similar transcriptional profile to both TGF-β isoforms (Fig. 2C).

Cross-tissue comparison of basal TGF-β levels revealed consistently higher TGF-β1 and TGF-β3 transcription in naïve OM and skin compared with VFM (*P*<0.01; Fig. 2D). Further, OM and skin both exhibited significantly greater increases in TGF-β1 transcription at 72 hours post-injury compared with VFM (*P*<0.01; Fig. 2E). TGF-β3 transcription increased significantly in VFM and OM at 72 hours post-injury but, notably, was unchanged in skin (Fig. 2F). Injury-induced TGF-β3 transcription in OM was significantly greater than in VFM and skin (*P*<0.01; Fig. 2F).

**Fig. 3:**
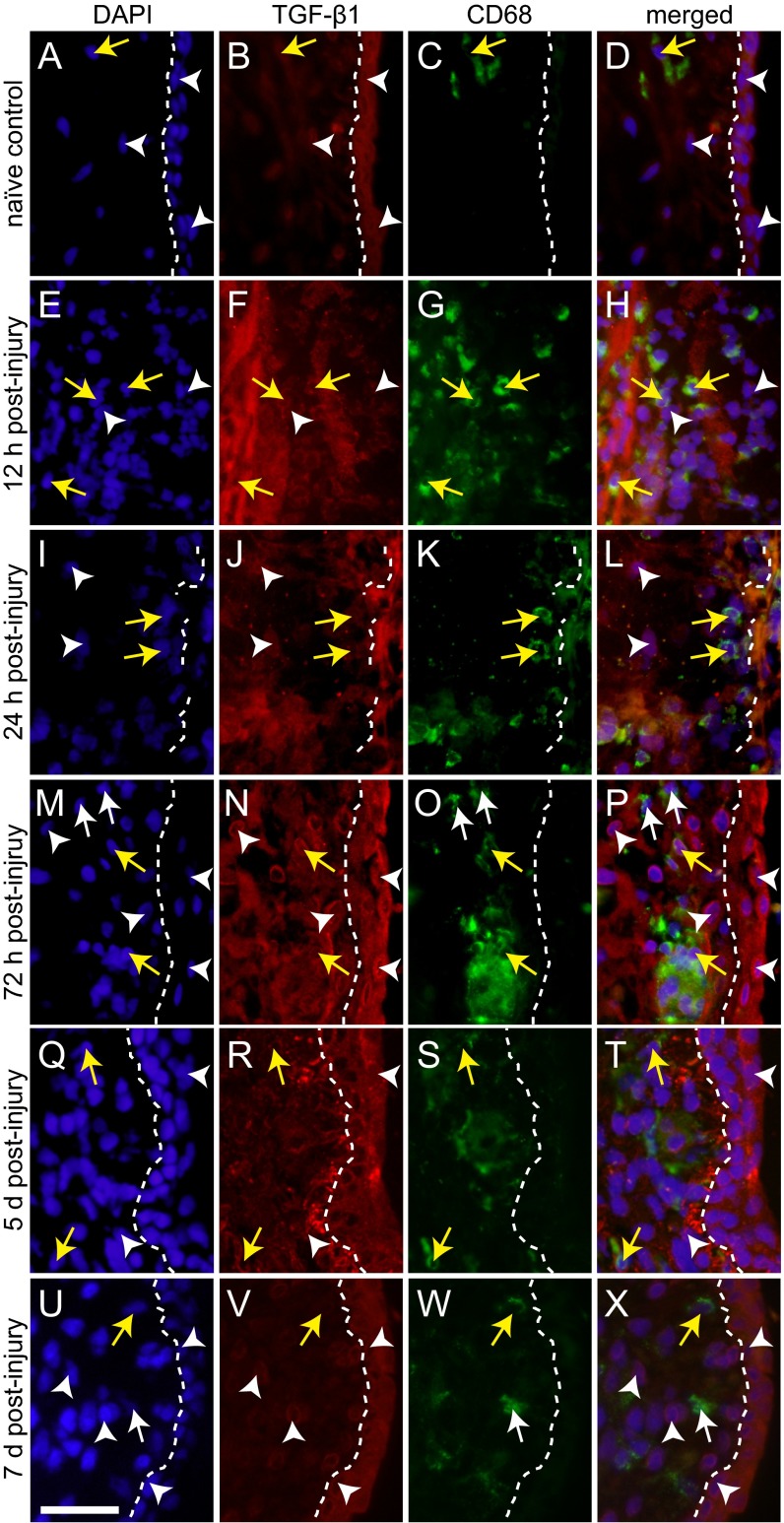
**TGF-β1 immunoactivity and CD68 colocalization in VFM post-injury.** (A-D) Representative images showing low TGF-β1 (red) and CD68 (green) immunosignals in naïve VFM. Nuclei are counterstained blue. (E-X) Representative images showing an increase in TGF-β1^+^ lamina propria and epithelial cells, and extracellular TGF-β1 immunosignals, 12 hours to 7 days post-injury. A subpopulation of the TGF-β1^+^ lamina propria cells are CD68^+^ macrophages. Each image reflects the stratified squamous cell region of the VFM and is representative of three independent animals and >3 replicate sections per animal. White arrowheads indicate TGF-β1^+^CD68^−^ cells; white arrows indicate TGF-β1^−^CD68^+^ macrophages; yellow arrows indicate TGF-β1^+^CD68^+^ macrophages; white dashed lines indicate the boundary between the lamina propria (left) and epithelium (right). The absence or fragmentation of these lines depicts incomplete reepithelialization. Scale bar: 30 μm.

**Fig. 4:**
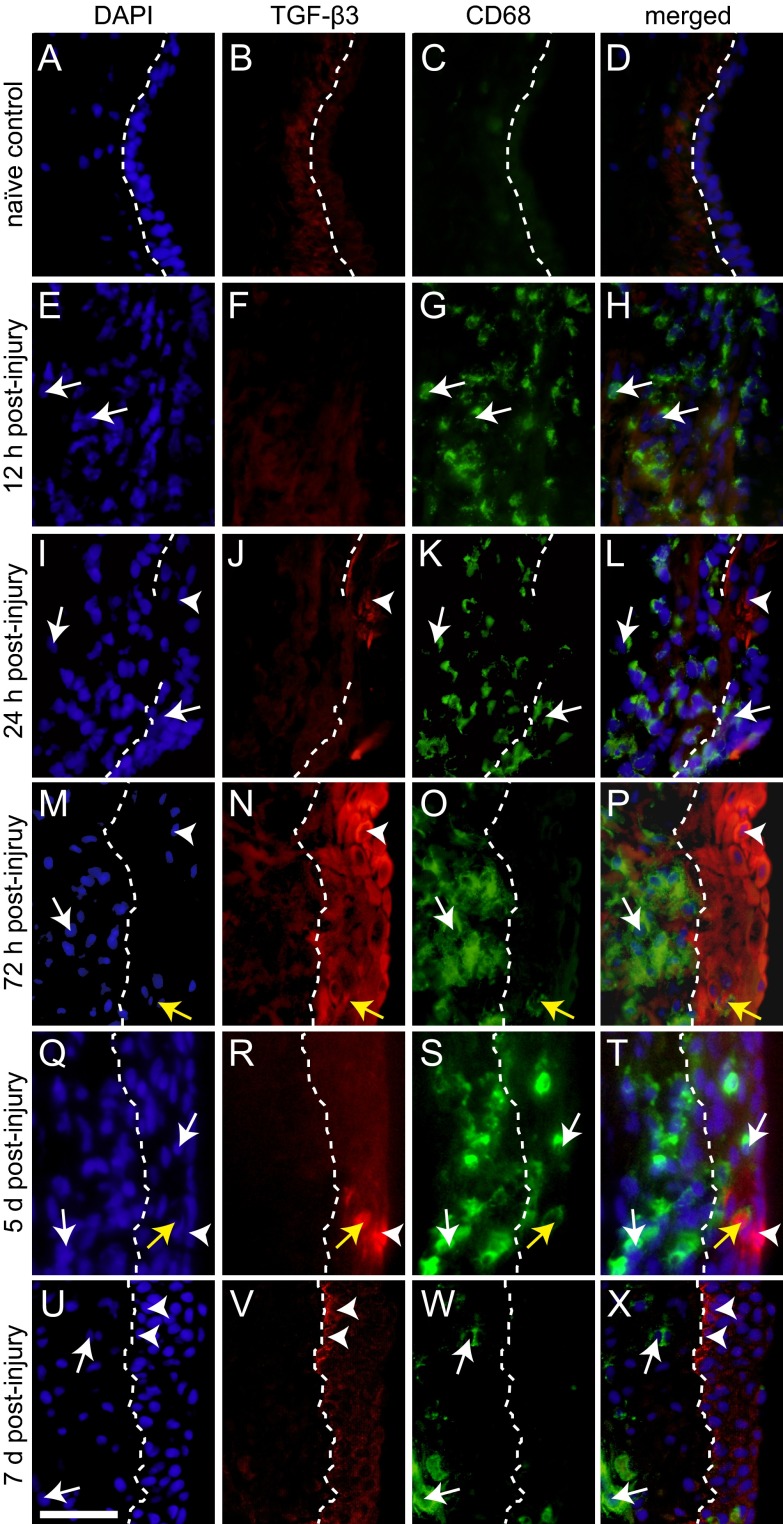
**Transient upregulation of TGF-β3 is associated with vocal fold epithelial cell recovery.** (A-D) Representative images showing low TGF-β3 (red) and CD68 (green) immunosignals in naïve VFM. Nuclei are counterstained blue. (E-X) Representative images showing initial appearance of TGF-β3 immunosignals corresponding to early epithelial cell recruitment at 24 hours post-injury, peak TGF-β3 expression corresponding to the completion of reepithelialization at 72 hours post-injury, and reduction of epithelial cell TGF-β3 expression at 5 and 7 days post-injury. The majority of TGF-β3^+^ cells are CD68^−^. Each image reflects the stratified squamous cell region of the VFM and is representative of three independent animals and >3 replicate sections per animal. White arrowheads indicate TGF-β3^+^CD68^−^ epithelial cells; white arrows indicate TGF-β3^−^CD68^+^ macrophages; yellow arrows indicate TGF-β3^+^CD68^+^ macrophages; white dashed lines indicate the boundary between the lamina propria (left) and epithelium (right). The absence or fragmentation of these lines depicts incomplete reepithelialization. Scale bar: 30 μm.

To corroborate these transcription data and evaluate protein-level expression by cell type and tissue region, we performed immunohistochemistry (IHC) against TGF-β1, TGF-β3 and macrophage marker CD68 (Damoiseaux et al., 1994). We observed significantly increased cellular and extracellular (secreted) TGF-β1 signals in injured VFM, beginning at 12 hours and continuing through 7 days post-injury. A subpopulation of TGF-β1^+^ cells in the wound bed at 12 hours were CD68^+^ macrophages (Fig. 3E–H). TGF-β1^+^ epithelial cells first appeared at 24 hours (Fig. 3I–L), were present through the completion of reepithelialization at 72 hours (Fig. 3M–P), and remained through 7 days post-injury (Fig. 3U–X). The recovering LP was populated by both TGF-β1^+^CD68^+^ and TGF-β1^+^CD68^−^ cells through 7 days post-injury (Fig. 3I–X). In contrast with TGF-β1, TGF-β3 was predominantly localized to the VF epithelium. TGF-β3 expression corresponded to early epithelial cell recruitment at 24 hours (Fig. 4I–L), peaked with the completion of reepithelialization at 72 hours (Fig. 4M–P), and decreased sharply at 5 days post-injury (Fig. 4Q–T). TGF-β3^+^CD68^+^ macrophages were occasionally identified within the recovering epithelium (Fig. 4M–T).

In naïve OM, TGF-β1 was expressed by the majority of epithelial cells (supplementary material Fig. S1A–C), whereas TGF-β3 was primarily localized to the basal cell layer (supplementary material Fig. S1G–I). Low intensity LP cell signals were observed for both TGF-β isoforms. Epithelial cells exhibited increased TGF-β1 and TGF-β3 expression at 72 hours post-injury, with a clear expression gradient at the wound edge – particularly for TGF-β3 (supplementary material Fig. S1J–L). In naïve skin, TGF-β1 and TGF-β3 were uniformly expressed throughout the epidermis and by a subpopulation of dermal cells (supplementary material Fig. S2A–C,G–I). Both TGF-β isoforms were expressed by recovering epidermal keratinocytes at 72 hours post-injury (supplementary material Fig. S2D–F,J–L); TGF-β3 did not exhibit an expression gradient comparable to OM.

### TGF-β3 is a less potent inducer of profibrotic molecule expression and myofibroblast differentiation than TGF-β1 and TGF-β2

**Fig. 5:**
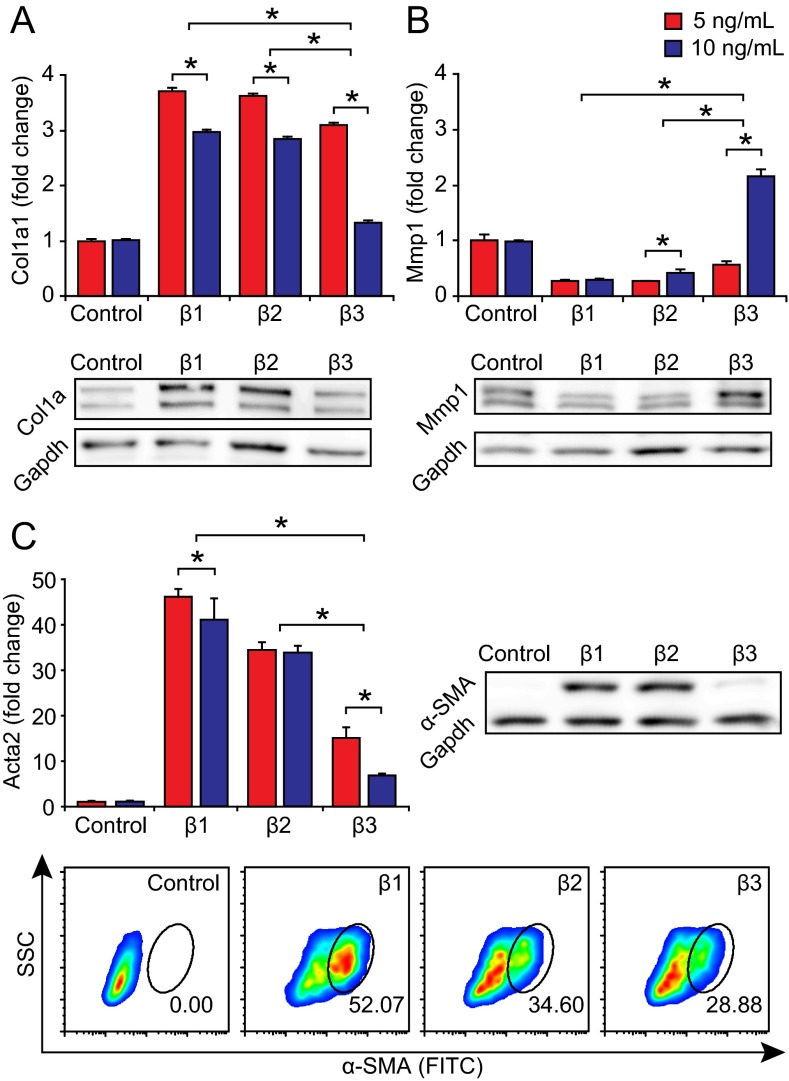
**TGF-β3 is a less potent inducer of profibrotic molecule expression and myofibroblast differentiation in human VFF than TGF-β1 and TGF-β2.** (A) Treatment with 5 and 10 ng/ml TGF-β3 induced less Col1a1 transcription than treatment with TGF-β1 or TGF-β2, in a dose-dependent manner. Western blotting was performed to evaluate collagen type 10 ng/ml condition. (B) Treatment with 5 and 10 ng/ml TGF-β3 induced greater Mmp1 transcription than treatment with TGF-β1 or TGF-β2, in a dose-dependent manner. Western blotting was performed to evaluate Mmp1 protein production in the 10 ng/ml condition. (C) Treatment with 5 and 10 ng/ml TGF-β3 induced less myofibroblast-specific marker Acta2 transcription than treatment with TGF-β1 or TGF-β2, in a dose-dependent manner. Western blotting and flow cytometry were performed to evaluate α-SMA protein expression and the α-SMA^+^ cell population in the 10 ng/ml condition. Results represent three independent experiments using primary human cells and are presented as fold change (means ± s.e.m.) relative to untreated controls (qRT-PCR) or representative images (western blots, flow cytometry plots). Separate control data for the 5 and 10 ng/ml conditions reflect separate qRT-PCR runs from the same experiment. Comparable data were obtained using an immortalized cell line. *P<0.01 for the TGF-β3 condition versus TGF-β1 or TGF-β2, and between doses for each isoform. All TGF-β treatment conditions exhibited significant differences compared with untreated controls for all genes of interest (for clarity of presentation, these comparisons are not denoted by an asterisk).

TGF-β1 is a potent inducer of fibrosis via both Smad-dependent and Smad-independent signaling pathways, contributing to increased fibroblast-myofibroblast differentiation, increased collagen production and reduced matrix metalloproteinase (Mmp) production (Derynck and Zhang, 2003; Zhang, 2009). Among the downstream molecules mediated by TGF-β, connective tissue growth factor (Ctgf/Ccn2) and endothelin 1 (Edn1) play an essential role in this fibrotic response (Leask, 2008; Leask et al., 2009). To gain insight into the therapeutic potential of TGF-β3 in VFM, we investigated whether this isoform differentially regulates these profibrotic molecules in cultured human VFFs, compared with TGF-β1 and TGF-β2. Treatment of primary human VFFs with 5 and 10 ng/ml TGF-β3 induced less Col1a1 transcription, greater Mmp1 transcription and less myofibroblast-specific marker Acta2 transcription than treatment with TGF-β1 or TGF-β2, in a dose-dependent manner (*P*<0.01; Fig. 5A–C). These transcript-level data were corroborated by western blotting and flow cytometry for the 10 ng/ml condition. Treatment with 10 ng/ml TGF-β3 also induced less Ctgf and Edn1 transcription than treatment with TGF-β1 or TGF-β2 (*P*<0.01; supplementary material Fig. S3). Finally, we evaluated TGF-β isoform cross-regulation in human VFF, based on reports of cross-regulation in dermal fibroblasts (O’Kane and Ferguson, 1997; Occleston et al., 2008a). Treatment with 10 ng/ml TGF-β3 induced significantly less endogenous transcription of TGF-β1 and TGF-β2, compared with treatment with TGF-β1 or TGF-β2 (*P*<0.01; supplementary material Fig. S4A,B). Interestingly, none of the isoforms induced endogenous transcription of TGF-β3 compared with control (supplementary material Fig. S4C). These results collectively demonstrate that TGF-β3 is a less potent inducer of key profibrotic molecule expression and fibroblast-myofibroblast differentiation in human VFF, compared with TGF-β1 and TGF-β2.

### TGF-β3 administration during the acute injury phase attenuates VFM scar formation *in vivo*

**Fig. 6:**
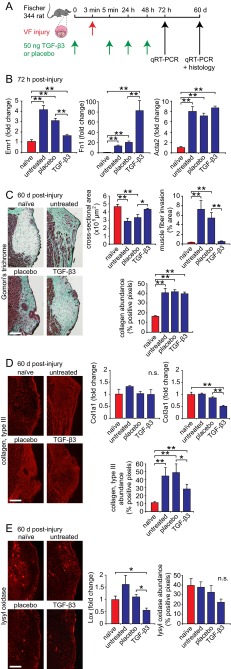
**TGF-β3 administration during the acute injury phase attenuates vocal fold scar formation *in vivo***. (A) Schematic showing experimental details. The red arrow indicates timing of vocal fold injury; green arrows indicate timing of TGF-β3 or placebo delivery; black arrows indicate experimental end points. (B) qRT-PCR data showing Emr1, Fn1 and Acta2 transcription in VFM at 72 hours post-injury (24 hours post-final TGF-β3 or placebo injection) in naïve, untreated, placebo-treated and TGF-β3-treated groups. (C) Representative Gomori’s trichrome-stained coronal sections at 60 days post-injury (left) and associated morphometric analysis of lamina propria cross-sectional area, muscle fiber invasion and collagen abundance (right). (D) Representative images showing immunostaining for collagen type III (left; red), Col1a1 and Col3a1 transcription (upper right), and morphometric analysis of collagen type III abundance (lower right), at 60 days post-injury. (E) Representative images showing immunostaining for lysyl oxidase (left; red), Lox transcription (center), and morphometric analysis of lysyl oxidase abundance (right), at 60 days post-injury. Results represent 3–5 independent animals per experimental group and time point and are presented as means ± s.e.m. **P<0.01 for all groups versus naïve, and for the TGF-β3-treated group versus placebo; *P<0.05 for all groups versus naïve, and for the TGF-β3-treated group versus placebo; n.s.=no significant difference.

Having demonstrated the muted effect of TGF-β3 (compared with TGF-β1 and TGF-β2) on profibrotic molecule expression *in vitro*, we next evaluated its therapeutic potential for scar attenuation in an established rat VFM injury model (Tateya et al., 2005). We pursued these *in vivo* experiments based on the hypothesis that exogenous TGF-β3 delivery could stimulate sufficient fibroblast activation and differentiation to achieve wound closure and maintain appropriate ECM synthesis, while protecting against the prolonged and excessive activation that is associated with fibrosis. We delivered 50 ng TGF-β3 or placebo 3 minutes prior to VFM injury creation, immediately post-injury, 24 hours post-injury and 48 hours post-injury (Fig. 6A). The dose was based on clinical trials in skin showing the greatest therapeutic effect at 500 ng TGF-β3 per linear centimeter of wound margin (Ferguson et al., 2009), adjusted to account for a rat membranous VFM length of 1 mm (Kurita et al., 1983). The treatment schedule was based on evidence that TGF-β3 therapy is effective when delivered both pre- and post-injury (Ferguson et al., 2009), and our earlier observation that endogenous TGF-β3 production does not peak until 72 hours post-injury (Fig. 4M–P). Quantitative RT-PCR-based evaluation of the wound site 72 hours post-injury revealed significantly less Emr1 transcription in the TGF-β3-treated group compared with the placebo-treated group (*P*<0.01; Fig. 6B), suggesting reduced macrophage infiltration at this time point. TGF-β3 treatment also induced significant upregulation of the ECM glycoprotein fibronectin (Fn1) (*P*<0.01; Fig. 6B), which acts to facilitate cell attachment and migration during early wound healing (Hirschi et al., 2002), but had no effect on Acta2 transcription compared with placebo (*P*>0.05; Fig. 6B). Evaluation of hyaluronic acid synthase transcription revealed significant upregulation of Has1 and Has2 in TGF-β3-treated VFM compared with placebo (*P*<0.05; supplementary material Fig. S5A), but no effect on Has3 (*P*>0.05; supplementary material Fig. S5A).

We evaluated fibrosis outcome at 60 days post-injury, based on earlier work demonstrating scar maturation at this time point in rat VFM (Tateya et al., 2005). TGF-β3-treated VFM exhibited LP morphology most similar to naïve control, with greater LP cross-sectional area (i.e. less tissue contraction; *P*<0.05; Fig. 6C) and reduced muscle fiber invasion (*P*<0.01; Fig. 6C) compared with placebo-treated VFM. Morphometric analysis of Gomori’s trichrome stain density revealed no significant difference in collagen abundance between TGF-β3- and placebo-treated groups (*P*>0.05; Fig. 6C); however, follow-up analyses of isoform-specific transcription showed no difference in Col1a1 but significantly reduced Col3a1 transcription in the TGF-β3-treated group compared with placebo (*P*<0.01; Fig. 6D). This finding was corroborated at the protein level using IHC with morphometric analysis of immunostain density (*P*<0.01; Fig. 6D). Evaluation of the collagen crosslinking molecule lysyl oxidase (Lox) revealed significantly reduced transcription in the TGF-β3-treated group compared with the placebo-treated group (*P*<0.05; Fig. 6E), but no significant difference at the protein level (*P*>0.05; Fig. 6E).

Follow-up analysis of hyaluronic acid synthase transcription at 60 days post-injury indicated reversal of the early-phase Has1 and Has2 upregulation identified at 72 hours (*P*<0.05; supplementary material Fig. S5B); however, morphometric analysis of Alcian Blue-stained sections (with hyaluronidase digestion control) showed greater hyaluronic acid accumulation in TGF-β3-treated VFM compared with placebo (*P*<0.01; supplementary material Fig. S5C), suggesting the possibility of transcriptional overcorrection at this time point.

## DISCUSSION

TGF-β1 and TGF-β3 have long been reported to hold differential effects on wound healing (Ferguson et al., 2009; Shah et al., 1995; Whitby and Ferguson, 1991), and possibly even account for cross-tissue differences in scar formation (Schrementi et al., 2008). Our data show, for the first time, that TGF-β1 and TGF-β3 are differentially expressed in naïve and acutely injured VFM, and in VFM compared with OM and skin. We also show that TGF-β3 is a less potent inducer of key profibrotic molecule expression and fibroblast-myofibroblast differentiation in human VFF, compared with TGF-β1 and TGF-β2; and that delivery of exogenous TGF-β3 during the acute injury phase modulates the inflammatory environment and reduces VFM scar formation *in vivo*. These findings corroborate and provide mechanistic support for a recent therapeutic trial using canine VFM (Ohno et al., 2012), as well as multiple reports in skin (Ferguson et al., 2009; McCollum et al., 2011; Shah et al., 1995; So et al., 2011), lip (Hosokawa et al., 2003) and OM (Ohno et al., 2011).

Our IHC data demonstrate that TGF-β1 and TGF-β3 are expressed by different cell types across different VFM tissue regions and exhibit different temporal expression patterns post-injury, suggesting that these two isoforms hold distinct roles in VFM maintenance and repair. TGF-β1 is expressed by VFFs and epithelial cells in both the SSC and CPF regions in naïve VFM, and appears to be delivered to the LP (in part) by CD68^+^ macrophages in the acute post-injury phase, consistent with reports in skin (Martin et al., 2003; Stramer et al., 2007). Once delivered to the LP, active TGF-β1 is available to stimulate VFF activation, myofibroblast differentiation and ECM synthesis, as demonstrated in our *in vitro* experiments. In contrast, TGF-β3 is predominantly expressed by CPF epithelial cells in naïve VFM and transiently expressed by SSC epithelial cells during active reepithelialization. It is rarely expressed within the LP region and is only occasionally expressed by CD68^+^ macrophages embedded in the epithelial milieu. These observations are consistent with findings in other systems that TGF-β3 directs epithelial cell proliferation and migration at the wound edge (Bandyopadhyay et al., 2006; Schrementi et al., 2008) and can accelerate wound closure when delivered therapeutically (Ohno et al., 2011).

We observed clear differences in the expression and localization of TGF-β1 and TGF-β3 in VFM, OM and skin, in both naïve and injury conditions. These post-injury comparisons were performed at 72 hours, as this time point marked peak TGF-β3 transcription and SSC epithelial cell immunolocalization in VFM. Our data are consistent with previous reports indicating that skin, which typically heals with scar, exhibits a high ratio of TGF-β1 to TGF-β3; whereas OM, which heals with minimal scar, and fetal tissue, which heals with no scar, exhibit significantly lower ratios of TGF-β1 to TGF-β3 (Cowin et al., 2001; Ferguson and O’Kane, 2004; Occleston et al., 2008a; Occleston et al., 2011; Schrementi et al., 2008). VFM, which can heal with scar, exhibits a different TGF-β1 and TGF-β3 transcriptional profile than both OM and skin, with less basal and injury-induced expression of both isoforms.

Our *in vivo* therapeutic trial involved repeated administration of TGF-β3 to the VFM, pre- and post-injury. This treatment schedule is consistent with: (i) the short half-life of TGF-β once activated (2–3 minutes in plasma) (Wakefield et al., 1990); (ii) a rationale that early TGF-β3 delivery might counteract the rapid release and activation of TGF-β1 by degranulating platelets post-injury (Blakytny et al., 2004) and that continued TGF-β3 delivery might counteract later TGF-β1 release by macrophages (Martin et al., 2003; Stramer et al., 2007); (iii) evidence of therapeutic benefit with repeated TGF-β3 administration in human skin trials (Ferguson et al., 2009; So et al., 2011); and (iv) evidence of therapeutic benefit with extended TGF-β3 bioavailability via genetic mutation (Waddington et al., 2010) or a sustained release delivery system (Manning et al., 2011). Our *in vivo* data show that repeated delivery of active TGF-β3 to the normally TGF-β1-predominant VFM injury site results in favorable tissue repair. A possible explanation, suggested by differences in competitive receptor binding ability among the TGF-β isoforms (Lyons et al., 1991), is that TGF-β3 acts as a TGF-β1 antagonist in the LP, successfully competing for the same receptor complex on resident VFFs and attenuating the transcription of downstream profibrotic molecules. Another possibility is that TGF-β3 reduces macrophage recruitment to the injury site directly (Occleston et al., 2011). We observed reduced Emr1 transcription following TGF-β3 treatment, inferring reduced macrophage infiltration and, by extension, reduced TGF-β1 trafficking. This finding is consistent with reports of reduced macrophage infiltration in scar-free fetal healing (Cowin et al., 1998) and TGF-β3-modulated skin repair (Shah et al., 1995). Finally, TGF-β3-treated VFM was marked by early upregulation of the ECM glycoprotein Fn1 and hyaluronic acid synthases Has1 and Has2, which again are overexpressed in fetal, compared with adult, skin wounds (Coolen et al., 2010; Mast et al., 1991) and have been shown to improve cell attachment, migration and eventual ECM remodeling (Clark, 1990; Martin, 1997).

TGF-β3 was a less potent stimulator of fibroblast-myofibroblast differentiation than TGF-β1 and TGF-β2 in our *in vitro* experiments, but had no effect on Acta2 transcription compared with the untreated and placebo-treated groups at 72 hours post-injury in our *in vivo* experiment. These findings are difficult to compare and reconcile because we did not deliver exogenous TGF-β1 and TGF-β2 *in vivo*, and because the TGF-β3-treated wound environment also contains endogenous TGF-β1 and possibly TGF-β2. Previous work has shown that sustained (7 days) infusion of TGF-β3 to uninjured skin results in less myofibroblast differentiation than equivalent infusion of TGF-β1 or TGF-β2 (Serini and Gabbiana, 1996), and that TGF-β3-treated lip and skin wounds contain fewer myofibroblasts than untreated wounds (Hosokawa et al., 2003; Waddington et al., 2010). It is well accepted that some level of myofibroblast activity is necessary for wound closure and the completion of reepithelialization, whereas persistent myofibroblast activity leading to excessive wound contraction and ECM synthesis is undesirable (Gurtner et al., 2008; Vyas et al., 2010). The lip and skin studies noted above evaluated myofibroblast accumulation by immunostaining for α-SMA^+^ cells between 72 hours and 14 days after TGF-β isoform delivery, and so it is reasonable to conclude that the Acta2 transcription event captured at 72 hours in our dataset does not represent the entire myofibroblast profile in injured and TGF-β3-treated VFM. It would therefore be helpful to evaluate the effect of TGF-β3 on persistent fibroblast-myofibroblast differentiation beyond the completion of VFM wound closure.

We observed favorable transcriptional and histological outcomes at 60 days post-injury, with TGF-β3-treated VFM exhibiting improved morphological appearance, reduced tissue contraction, reduced Col3a1 transcription, reduced collagen type III protein abundance and increased hyaluronic acid abundance, compared with placebo-treated VFM. Collagen type III is important for tissue extension and elasticity and is the most abundant collagen isoform in naïve rat and human VFM (Tateya et al., 2005; Tateya et al., 2006a), as well as in chronically scarred rat VFM (Tateya et al., 2005), supporting its relevance as a marker of fibrotic outcome in this tissue. Our observation of improved tissue morphology without muscle fiber invasion of the LP suggests that TGF-β3 helps facilitate appropriate positional signaling and morphologic patterning during wound repair. A recent study in OM noted a similar finding following TGF-β3 administration, with less fusion of the treated mucosa and its underlying muscle (Ohno et al., 2011). Normalization of tissue morphology has also been reported in TGF-β3-treated skin injuries, where remodeled collagen appears in basket-weave formation, similar to uninjured skin (Ferguson et al., 2009; Occleston et al., 2009; Occleston et al., 2011).

In summary, our data show that TGF-β3 redirects wound healing to reduce VFM scar formation *in vivo*. Taken together, this study and evidence from the wider literature suggest that TGF-β3 exerts its effects on multiple phases of the tissue repair process: (i) as a TGF-β1 antagonist, (ii) by modulating fibroblast-myofibroblast differentiation, (iii) by facilitating cell migration and accelerating reepithelialization, (iv) by interrupting macrophage recruitment, (v) by restricting profibrotic gene transcription and (vi) by directing ECM synthesis, remodeling and morphologic patterning. Future work in VFM should examine these putative mechanisms in more detail and, with a view to clinical translation, evaluate optimal dose, delivery methods and the effect on biomechanical tissue performance and voice restoration.

## MATERIALS AND METHODS

### Animal procedures and tissue harvest

Four-month-old Fischer 344 male rats (Charles River, Wilmington, MA) were used in all experiments. Rat VFM shares many anatomic, cellular and extracellular features with human VFM and is well established as an injury model (Ling et al., 2010; Tateya et al., 2005; Tateya et al., 2006b); however, it is not subject to the same biomechanical forces that occur during human phonation (Riede, 2011; Riede et al., 2011). All experimental procedures were performed in accordance with the Public Health Service Policy on Humane Care and Use of Laboratory Animals, and the Animal Welfare Act (7 U.S.C. et seq.); the animal use protocol was approved by the Institutional Animal Care and Use Committee (IACUC) of the University of Wisconsin-Madison.

Animals in the TGF-β isoform expression experiments received a bilateral (qRT-PCR) or unilateral (histology and IHC) VFM stripping injury under endoscopic guidance (Tateya et al., 2005), a 2-cm OM incision injury (qRT-PCR, histology and IHC) or a 2-cm skin incision injury (qRT-PCR, histology and IHC). A bilateral VFM injury was required to obtain sufficient total RNA for downstream qRT-PCR, whereas a unilateral VFM injury was used for histology and IHC to preserve the contralateral VFM as an additional within-animal control. Rats were euthanized and tissue harvested at 12 hours and 1, 3, 5 and 7 days post-injury (VFM; *n*=5 per time point for qRT-PCR, *n*=3 per time point for histology and IHC) and 3 days post-injury (OM and skin; *n*=5 per time point for qRT-PCR, *n*=3 per time point for histology and IHC). Experimentally naïve control rats were age-matched to the 7 days (for VFM) or 3 days (for OM and skin) post-injury time points.

Animals in the TGF-β3 treatment experiment were divided into four groups (*n*=8-10 per group): naïve control; bilateral VFM injury with no treatment; bilateral VFM injury with placebo treatment (4 mM HCl carrier solution); bilateral VFM injury with TGF-β3 treatment (50 ng/μl TGF-β3 in 4 mM HCl; R&D Systems, Minneapolis, MN). TGF-β3 (1 μl, 50 ng) or placebo was injected 3 minutes before bilateral VFM injury creation, immediately post-injury (~2 minutes following the first injection), 24 hours post-injury and 48 hours post-injury. VFM injections targeted the intact LP (pre-injury) or residual LP/wound bed (post-injury) and were performed under endoscopic guidance using a 50-mm, 26-gauge needle and 5-μl syringe (SGE Analytical Science, Melbourne, Australia) (Welham et al., 2008). Rats were euthanized and tissue harvested at 72 hours (*n*=5 per group for qRT-PCR) and 60 days (*n*=4-5 per group for qRT-PCR, histology and IHC) post-injury.

Tissue samples intended for RNA extraction were microdissected in an RNase-free environment, placed in 350 μl TLR buffer (Qiagen, Valencia, CA) on ice and stored at −80°C until RNA isolation. Samples intended for histology and IHC were embedded in Optimum Cutting Temperature compound (Tissue-Tek; Sakura, Tokyo, Japan). Frozen sections of 8 μm thickness were prepared in the coronal plane using a cryostat.

### Histology and IHC

Histology and immunostaining were performed using midmembranous VFM coronal sections at the level of the larygneal alar cartilage, as previously described (Ling et al., 2010). Cell and tissue morphology were evaluated using hematoxylin and eosin (H&E); collagen distribution was evaluated using Gomori’s trichrome; and hyaluronic acid distribution was evaluated using Alcian Blue (pH 2.5) staining of adjacent sections, with and without hyaluronidase digestion (37°C for 2 hours). Sections intended for IHC were fixed using either 2% paraformaldehyde or acetone for 10 minutes, washed with PBS and blocked using 5% BSA (Sigma, St Louis, MO) for 60 minutes. Sections were then sequentially incubated with a primary antibody for 90 minutes, a relevant secondary antibody for 60 minutes, and counterstained with DAPI (MP Biomedicals, Santa Ana, CA). For both single- and double-immunostaining, control sections stained with an isotype control, or without the primary or secondary antibody, ensured that each signal was specific to the intended antigen (supplementary material Fig. S6).

The primary antibodies used were: rabbit anti-TGF-β1 (1:250) (Flanders et al., 1989); rabbit anti-TGF-β3 (1:150) (Flanders et al., 1991); mouse anti-CD68, clone ED1 (1:750; AbD Serotec, Raleigh, NC) rabbit anti-collagen, type III (1:50; Rockland Immunochemicals, Gilbertsville, PA); and goat anti-lysyl oxidase (1:50; Santa Cruz Biotechnology, Santa Cruz, CA). The secondary antibodies used were: Alexa Fluor 488 goat anti-mouse IgG, Alexa Fluor 594 goat anti-rabbit IgG and Alexa Fluor 555 donkey anti-goat IgG (1:600; Invitrogen, Carlsbad, CA).

Microscopy was performed using a microscope with both brightfield and fluorescent capabilities (E-600; Nikon, Melville, NY) equipped with a digital microscopy camera (DP-71; Olympus, Center Valley, PA). All images intended for quantitative analysis were captured with consistent exposure settings. MetaMorph 7.5 (Molecular Devices, Downingtown, PA) was used to quantify LP cross-sectional area and LP protein abundance, as previously described (Yamashita et al., 2010). Threshold values were established using the HSI color model for Gormori’s trichrome- and Alcian Blue-stained images, and the RGB color model for immunostained images. Threshold values were applied consistently across all samples.

### Cell culture

Primary and immortalized human VFFs (Chen and Thibeault, 2009) were maintained in DMEM (Sigma, St Louis, MO), supplemented with 10% fetal bovine serum (Sigma, St Louis, MO), 100 U/ml penicillin, 100 μg/ml streptomycin, 0.25 μg/m amphotericin B (Invitrogen) and 1% NEAA (Sigma). For all TGF-β treatment experiments, passage 3 primary cells or passage 8 immortalized cells were plated at 4×10^4^ cells/well density in six-well plates and cultured until 70% confluent. After 24 hours of serum starvation, cells were treated with or without 5 or 10 ng/ml TGF-β1, TGF-β2 or TGF-β3 (R&D Systems) and harvested at 24 hours for qRT-PCR, 48 hours for western blotting and 72 hours for flow cytometry.

### RNA isolation and RT-PCR

Total RNA was isolated using the RNeasy Micro Plus kit (Qiagen) according to the manufacturer’s instructions. RNA yield and integrity were first evaluated using a NanoDrop ND-1000 spectrophotometer (NanoDrop, Wilmington, DE). Samples with a concentration above 20 ng/ml and OD_260:280_ of 1.8–2.0 were further evaluated using 1% agarose gel electrophoresis. Samples with no evidence of RNA degradation were retained. Reverse transcription was performed using the iScript cDNA Synthesis kit (Bio-Rad, Hercules, CA) with 10 or 20 ng input total RNA per 20 μl reaction, according to the manufacturer’s instructions. Negative control samples were performed in parallel by omitting RNA template or reverse transcriptase.

qRT-PCR was performed on a 7500 Fast Real-Time PCR system (Applied Biosystems, Foster City, CA) using the QuantiTect SYBR Green PCR kit (Qiagen). Each 25 μl total volume reaction contained 12.5 μl 2× QuantiTect master mix, 2.5 μl 10× QuantiTect primer assay and 10 μl cDNA template diluted in nuclease-free H_2_O. Amplifications were performed in MicroAmp Fast Optical 96-well reaction plates with optical adhesive film covers (Applied Biosystems) according to cycling conditions suggested for the Applied Biosystems 7500 instrument in the QuantiTect SYBR Green handbook (initial activation at 95°C for 15 minutes; 40 cycles of 94°C for 15 seconds, 55°C for 30 seconds, 72°C for 30 seconds). All samples were run in duplicate (animal samples) or triplicate (human VFF samples) using cDNA synthesized from the same batch and starting amount of total RNA. Negative controls containing no cDNA template were included for each gene within each PCR run. To avoid variation in amplification conditions across runs, reactions for all experimental conditions (i.e. all non-injury controls and post-injury time points) for each gene of interest were performed in the same 96-well plate. Amplification specificity for each gene was confirmed by a single distinct melting curve, followed by 1.5% agarose gel electrophoresis of PCR products to confirm the presence of a single band at the expected amplicon size. Relative mRNA expression was calculated using the 2^−ΔΔCT^ method (Livak and Schmittgen, 2001) and normalized against previously validated housekeeping genes (Tbp, Sdha and Wrnip1) (Chang et al., 2010).

The following commercial rat- or human-specific primers (QuantiTect, Qiagen) were used for PCR amplification: QT00370622 (rat Col1a1); QT01083537 (rat Col3a1); QT01615901 (rat Acta2); QT00391272 (rat Emr1); QT00179333 (rat Fn1); QT01169448 (rat Has1); QT00194537 (rat Has2); QT00192857 (rat Has3); QT00185591 (rat Lox); QT00187796 (rat TGF-β1); QT00187796 (rat TGF-β3); QT00195958 (rat Sdha); QT00183344 (rat Wrnip1); QT00088102 (human Acta2); QT00052899 (human Ctgf); QT00037793 (human Col1a1); QT00088235 (human Edn1); QT00014581 (human Mmp1); QT00000721 (human Tbp); QT00000728 (human TGF-β1); QT00025718 (human TGF-β2); and QT00001302 (human TGF-β3).

### Western blotting

Whole-cell lysates were prepared in RIPA buffer (Pierce Biotechnology, Rockford, IL) for 15 minutes on ice. Reducing SDS-PAGE was performed using a pre-cast 10% acrylamide gel (Bio-Rad) with 10 or 20 μg total protein load. Following transfer, polyvinylidene fluoride membranes were blotted using the following primary antibodies: mouse anti-α-SMA, clone 1A4 (1:1000; Sigma); goat anti-collagen, type I (1:400; Southern Biotechnology, Birmingham, AL); mouse anti-Mmp1 (1:500; Southern Biotechnology); and mouse anti-Gapdh (1:5000; Sigma). Blots were detected using the Immun-Star WesternC Chemiluminescence kit (Bio-Rad) with relevant horseradish peroxidase (HRP)-conjugated anti-mouse and anti-goat IgG secondary antibodies (1:5000), according to the manufacturer’s instructions.

### Flow cytometry

Single-cell suspensions were fixed and permeabilized with fixation/permeabilization solution (eBioscience, San Diego, CA) for intracellular staining. Cells were incubated with FITC-conjugated anti-α-SMA, clone 1A4 (1:125; Sigma) for 30 minutes. Flow cytometry data were acquired using a FACSCalibur instrument and CellQuest software (Becton Dickinson, Franklin Lakes, NJ). Additional analysis was performed using FlowJo software (Tree Star, Ashland, OR). Gates (cutoffs) were set according to unstained controls.

### Data analysis

*In vivo* qRT-PCR and morphometric data were analyzed using one-way analysis of variance (ANOVA). *In vitro* TGF-β isoform treatment data were analyzed using two-way ANOVA, with TGF-β isoform and treatment dose as fixed effects, and their interaction term included. Data were rank-transformed where needed to meet the equal variance assumptions of ANOVA. In all ANOVA models, if the omnibus *F* test revealed a significant difference, planned pairwise comparisons (specified in each relevant figure caption) were performed using Fisher’s protected least significant difference method. All *P*-values were two-sided.

## Supplementary Material

Supplementary Material
