# Supplementary material for: TGF-β3 modulates the inflammatory environment and reduces scar formation following vocal fold mucosal injury in rats
Source: Dis Model Mech. 2013 Oct 2;7(1):83–91. doi: 10.1242/dmm.013326 (PMC3882051; doi:10.1242/dmm.013326)
Supplement: Supplementary Material [file supp_7_1_83__index.html]

TGF-β3 modulates the inflammatory environment and reduces scar formation following vocal fold mucosal injury in rats — TGF-β3 modulates the inflammatory environment and reduces scar formation following vocal fold mucosal injury in rats — Supplementary Material 

# TGF-β3 modulates the inflammatory environment and reduces scar formation following vocal fold mucosal injury in rats

## DMM013326 Supplementary Material

**Files in this Data Supplement:**

- **Supplementary Material PDF**
