# Supplementary material for: TGF-β3 modulates the inflammatory environment and reduces scar formation following vocal fold mucosal injury in rats
Source: Dis Model Mech. 2013 Oct 2;7(1):83–91. doi: 10.1242/dmm.013326 (PMC3882051; doi:10.1242/dmm.013326)
Supplement: Supplementary Material [file supp_013326_DMM013326.pdf]

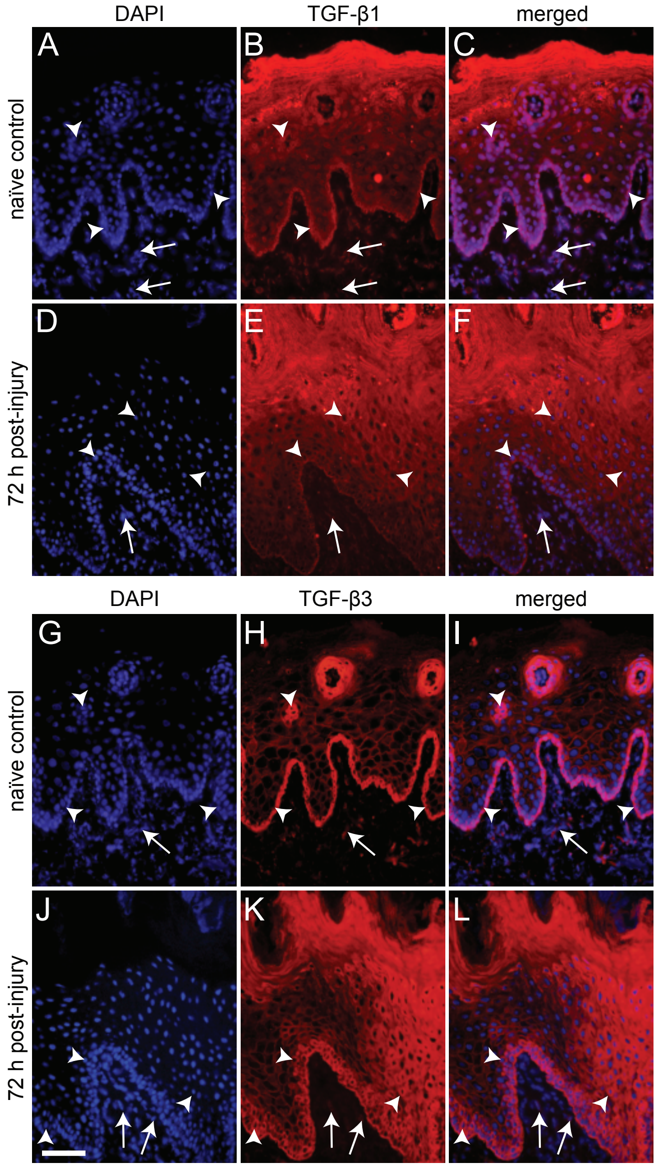

Figure S1. TGF-β1 and β3 immunoactivity in naïve and injured oral mucosa. (A-F) Representative images showing TGF-β1 immunosignals (red) in naïve oral mucosa and at 72 h post-injury. Nuclei are counterstained blue. White arrows indicate TGF-β1$^+$ lamina propria cells. White arrowheads indicate TGF-β1$^+$ epithelial cells. (G-L) Representative images showing TGF-β3 immunosignals (red) in naïve oral mucosa and at 72 h post-injury. White arrows indicate TGF-β3$^+$ lamina propria cells. White arrowheads indicate TGF-β3$^+$ epithelial cells. Each image is representative of 3 independent animals and >3 replicate sections per animal. The wound edge is towards the right of each panel. Scale bar = 30 μm.

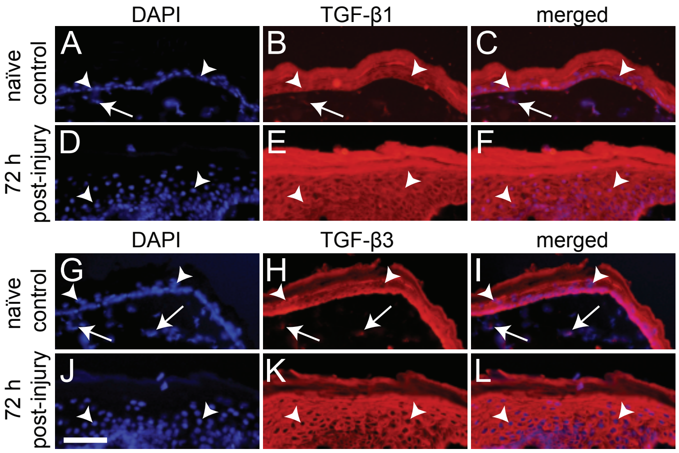

Figure S2. TGF-β1 and β3 immunoactivity in naïve and injured skin. (A-F) Representative images showing TGF-β1 immunosignals (red) in naïve skin and at 72 h post-injury. Nuclei are counterstained blue. White arrows indicate TGF-β1^{+} dermal cells. White arrowheads indicate TGF-β1^{+} keratinocytes. (G-L) Representative images showing TGF-β3 immunosignals (red) in naïve skin and at 72 h post-injury. White arrows indicate TGF-β3^{+} dermal cells. White arrowheads indicate TGF-β3^{+} keratinocytes. Each image is representative of 3 independent animals and >3 replicate sections per animal. The wound edge is towards the right of each panel. Scale bar = 30 μm.

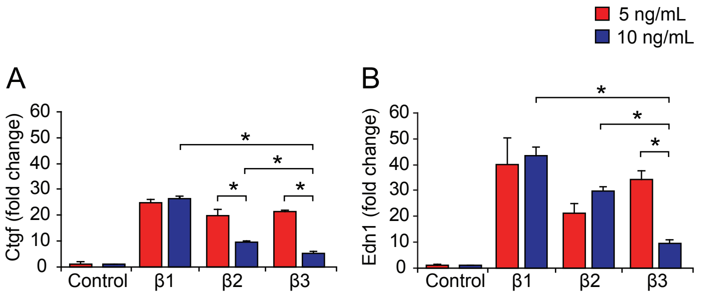

Figure S3. TGF-β3 is a less potent inducer of Ctgf and Edn1 gene expression in human vocal fold fibroblasts, compared to TGF-β1 and β2. (A) Treatment with 10 ng/mL TGF-β3 induced less Ctgf transcription than treatment with TGF-β1 or β2. (B) Treatment with 10 ng/mL TGF-β3 induced less Edn1 transcription than treatment with TGF-β1 or β2. Results represent 3 independent experiments using primary human cells and are presented as mean fold change ± SE relative to untreated controls. Separate control data for the 5 and 10 ng/mL conditions reflect separate qRT-PCR runs from the same experiment. Comparable data were observed using an immortalized cell line. * = $p < 0.01$ for the TGF-β3 condition versus TGF-β1 or β2, and between doses for each isoform. All TGF-β treatment conditions exhibited significant differences compared to untreated controls for both genes of interest (for clarity of presentation, these comparisons are not denoted by *).

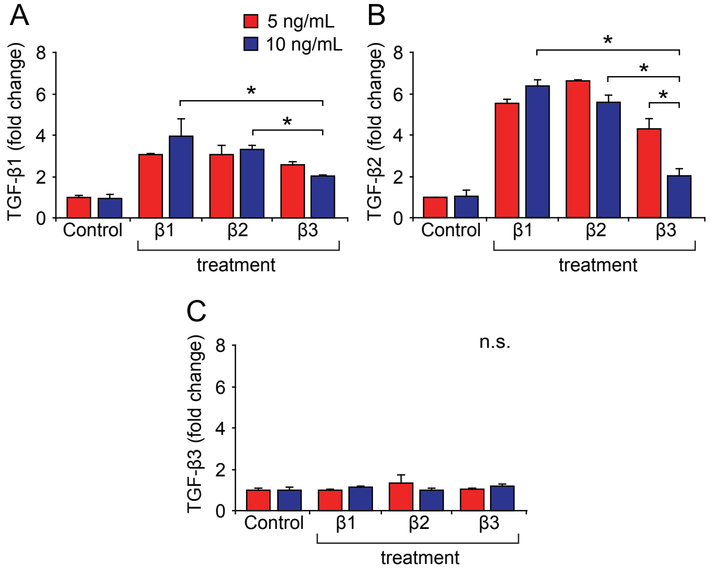

Figure S4. TGF-β3 demonstrates moderate cross-regulation of endogenous TGF-β1 and β2 expression in human vocal fold fibroblasts. (A) Treatment with 10 ng/mL TGF-β3 induced less endogenous TGF-β1 transcription than treatment with TGF-β1 or β2. (B) Treatment with 10 ng/mL TGF-β3 induced less endogenous TGF-β2 transcription than treatment with TGF-β1 or β2. (C) Treatment with TGF-β1, β2 or β3 had no effect on endogenous TGF-β3 transcription. Results represent 3 independent experiments using primary human cells and are presented as mean fold change ± SE relative to untreated controls. Separate control data for the 5 and 10 ng/mL conditions reflect separate qRT-PCR runs from the same experiment. Comparable data were observed using an immortalized cell line. * = $p < 0.01$ for the TGF-β3 condition versus TGF-β1 or β2, and between doses for each isoform; n.s. = no significant differences. All TGF-β treatment conditions in panels A and B exhibited significant differences compared to untreated controls for both genes of interest (for clarity of presentation, these comparisons are not denoted by *).

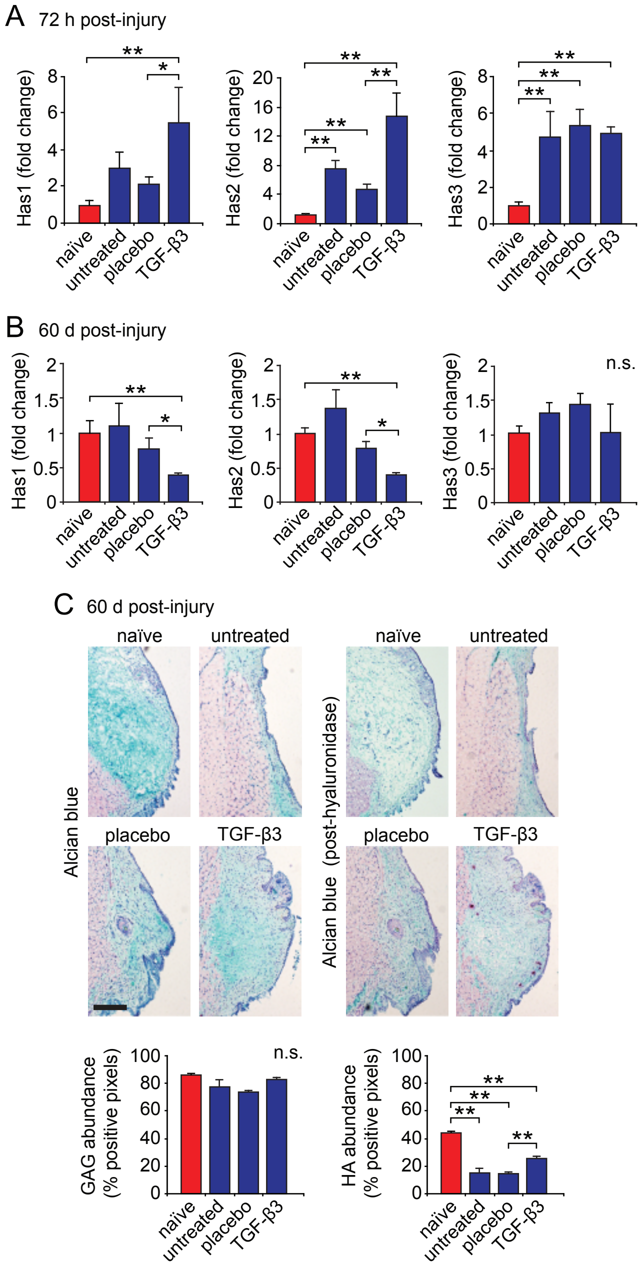

Figure S5. TGF-β3 administration during the acute injury phase alters early/late Has transcription and eventual hyaluronic acid abundance in vivo. (A) qRT-PCR data showing Has1, Has2 and Has3 transcription in vocal fold mucosa at 72 h post-injury (24 h post-final TGF-β3/placebo injection) in naïve, untreated, placebo-treated and TGF-β3-treated groups. (B) Comparable qRT-PCR data at 60 d post-injury. (C) Representative Alcian blue-stained coronal sections at 60 d post-injury and associated morphometric analysis of mucopolysaccharide/glycosaminoglycan (GAG) and hyaluronic acid (HA) abundance. HA abundance was calculated by comparing signal intensity in adjacent (8 μm) sections with and without hyaluronidase digestion. Results represent 3-5 independent animals per experimental group and time point and are presented as mean ± SE. ** = $p < 0.01$ for all groups versus naïve, and for the TGF-β3-treated group versus placebo; * = $p < 0.05$ for all groups versus naïve, and for the TGF-β3-treated group versus placebo; n.s. = no significant differences.

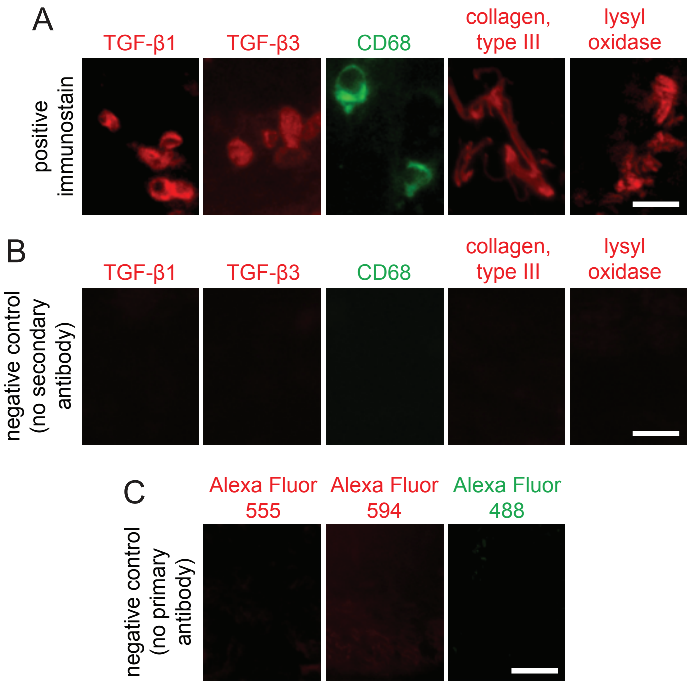

Figure S6. Immunostaining controls. (A) Representative high-magnification images of rat vocal fold lamina propria showing cell-specific immunostaining patterns for the TGF-β1, TGF-β3 and CD68 primary antibodies, and matrix-specific staining patterns for the collagen, type III and lysyl oxidase primary antibodies used in this study. (B) Representative negative control images for the complete immunostaining protocol with omission of the secondary antibody incubation step. (C) Representative negative control images for the complete immunostaining protocol with omission of the primary antibody incubation step. Additional positive and negative control data for anti-TGF-β1 and anti-TGF-β3, the two non-commercial antibodies used in this study, have been previously reported (Flanders et al., 1989, 1991). Scale bar = 10 μm.
